# Microbiological profile of bloodstream infections and antimicrobial resistance patterns at a tertiary referral hospital in Amazon, Brazil

**DOI:** 10.1590/0037-8682-0382-2023

**Published:** 2023-09-22

**Authors:** Ewerton da Silva Ferreira, Aline Stephanie Pérez Gómez, Taynná Vernalha Rocha Almeida, Carlos Henrique Michiles Frank, Sabrina Araújo de Melo, Eveny Perlize Melo Marinho, Sergio Damasceno Pinto, Pablo Vinicius Silveira Feitoza, Rossicleia Lins Monte, Michele de Souza Bastos

**Affiliations:** 1 Fundação de Medicina Tropical Doutor Heitor Vieira Dourado, Laboratório de Bacteriologia, Manaus, AM, Brasil.; 2 Universidade Federal do Amazonas, Programa de Pós-graduação em Biotecnologia, Manaus, AM, Brasil.; 3 Universidade Federal do Amazonas, Programa de Pós-graduação em Ciências da Saúde, Manaus, AM, Brasil.

**Keywords:** Antimicrobial resistance, Bloodstream infections, Sepsis, MRSA, ESBL

## Abstract

**Background:**

Bloodstream infections (BSI) are a global health issue, leading to high mortality and morbidity among hospitalized patients.

**Methods:**

A retrospective, observational and descriptive study was conducted by reviewing blood culture records collected from patients with suspected BSI, between January 2017 and December 2019.

**Results:**

The most frequent antimicrobial resistant (AMR) pathogens were methicillin-resistant*Staphylococcus aureus*(MRSA) (40%), methicillin-resistant*S. epidermidis* (MRSE) (9.5%), and extended-spectrum beta-lactamase (ESBL)-producing *Enterobacteriaceae* (35.3%).

**Conclusions:**

Our findings underscore the importance of continued vigilance and advocate for the rational use of antimicrobial agents.

Bloodstream infections (BSI) constitute a significant global health issue due to their contribution to high mortality rates among hospitalized patients. Untreated or incorrectly treated infections can lead to sepsis, septic shock, organ failure, and death. Globally, sepsis affects approximately 50 million people, resulting in approximately 11 million deaths yearly^1^.

Human immune cells are responsible for fighting the pathogenic invasions that cause sepsis. However, immunocompromised hosts cannot mount an effective immune response, heightening the risk of death^2^. Thus, people living with human immunodeficiency virus (HIV) / acquired immunodeficiency syndrome (AIDS) (designated PLWHA) are at an increased risk of BSI. Even after reaching a stable immune status, PLWHA represent a susceptible population. This is primarily due to their prolonged hospitalization times, especially in intensive care units, owing to secondary conditions^3^.

Due to the high incidence and global mortality associated with BSIs, it is essential to monitor changes in antimicrobial resistance (AMR) patterns. Integrated data on pathogen circulation and resistance profiles is needed for the establishment of an effective prevention and surveillance network. The primary objective of this study was to investigate the clinical and demographic aspects of patients with BSI and to associate them with the AMR profiles of common pathogens in blood cultures. To this end, samples were collected from a tertiary hospital in Manaus, Amazonas, Brazil. 

We conducted a retrospective, observational, and descriptive study that reviewed the blood culture records of patients admitted to the Fundação de Medicina Tropical Dr. Heitor Vieira Dourado (FMT-HVD) between January 2017 and December 2019. Clinical, demographic, and laboratory data were anonymized from individual medical records. Positive blood cultures from all patients enrolled in the FMT-HVD study were included, and the first result was considered for those who underwent the test more than once. We considered only *S. epidermidis* to be the accurate diagnosis when two blood cultures were positive with one or more clinical signs of infection. Some of the patients were PLWHA and were enrolled in the sexually transmitted infections program at this center.

Blood samples from 3,795 suspected cases of BSI were collected under aseptic conditions. The bacteriology laboratory used the BD Bactec 9240 system (Becton, Dickinson and Company, NJ, USA) in conjunction with BD BACTEC Plus Aerobic medium (Becton Dickinson). Gram stain, agar culture (blood agar, chocolate agar, and EMB agar), and biochemical tests confirmed the identification of the respective bacteria. The Kirby-Bauer disk diffusion method was used for antimicrobial susceptibility testing. For the identification of methicillin-resistant *Staphylococcus* sp., the cefoxitin test was performed according to the Clinical and Laboratory Standards Institute (CLSI-2022/2023)^4^. Enterobacteriaceae that produced extended-spectrum beta-lactamases (ESBL) were identified using the commercial Gram-negative MicroScan panel (Beckman Coulter, Brea, CA, USA). This phenotypic test provided both microbial identification and insights into bacterial resistance mechanisms based on probabilistic data. Strains were categorized as multidrug-resistant (MDR) if they exhibited resistance to one antimicrobial agent in three or more antimicrobial categories^5^. We followed the checklist of the Microbiology Investigation Criteria for Reporting Objectively (Supplementary data)^6^. 

For statistical analysis, patients were classified into two groups: methicillin-resistant SA and methicillin-susceptible SA. For descriptive analysis, absolute and relative frequencies as well as odds ratios (OR) with 95% (CI95%) were calculated to compare categorical variables. The OR was considered statistically different from 1 if p < 0.05. The study was approved by the Research Ethics Committee (CEP/FMT-HVD) following the regulations for research on humans, under number 3471750; CAAE: 154687 19.8.0000.0005.

Of the 3,795 blood specimens, 659 (17.3%) tested positive for microorganisms. We excluded 169 samples (4.4%) from the analysis due to their positivity for other agents, such as fungi and mycobacteria. Thus, a total of 490 blood cultures (74.3%; 490/659) were analyzed. Among these, 68.9% (338/490) of the patients were men, 47.9% (235/490) were aged between 19-44 years, and 37.3% (183/490) were PLWHA. Gram-positive cocci represented 70.6% (346/490) of isolates. Specifically, 17.3% (60/490) were identified as *S. aureus*, and 21.1% (73/490) as *S. epidermidis.* Among these, 40% (24/60) and 9.5% (7/73) were methicillin-resistant, respectively (Figure 1). 

**FIGURE 1:**
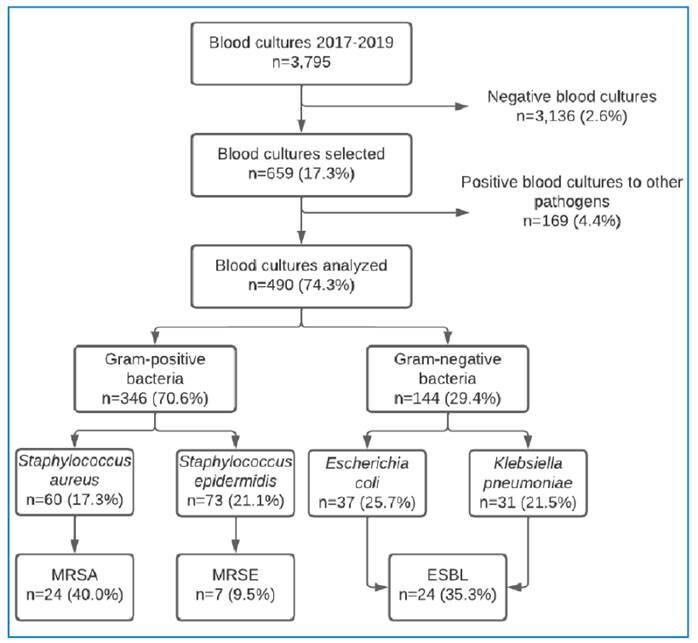
Flowchart of positive blood cultures at the Fundação de Medicina Tropical Doutor Heitor Vieira Dourado, from January 2017 to December 2019.

The antimicrobial resistance profiles for methicillin-resistant *Staphylococcus sp.* (1a), and ESBL-producers *Enterobacteriaceae* (1b) are presented in Table 1. We noted high resistance rates to several antimicrobial agents; however, none of the MRSA isolates displayed resistance to vancomycin or linezolid. *Enterobacteriaceae* emerged as the most frequently isolated gram-negative organisms, accounting for 29.4% (144/490) of the total, with *Escherichia coli* representing 25.7% (37/144) and *Klebsiella pneumoniae* comprising 21.5% (31/144). Out of the combined 68 isolates of these two species, 35.3% (24/68) exhibited the ESBL phenotype (9 *E. coli* and 15 *K. pneumoniae* isolates). We observed that *E. coli* ESBL was more resistant to the other non-beta-lactam antimicrobial agents when compared to *K. pneumoniae* ESBL. MDR strains were not detected in this study.

**TABLE 1A:** Resistance profile of MRSA and MRSE isolates.

Antimicrobial drug	MRSA^a^ resistance (n = 24) (%)	MRSE^b^ resistance (n = 7) (%)
Clindamycin	20 (83.3%)	7 (100%)
Erythromycin	20 (83.3%)	7 (100%)
Ciprofloxacin	16 (66.6%)	6 (85.7%)
Gentamicin	15 (62.5%)	6 (85.7%)

^a^ **MRSA:** Methicillin resistant *Staphylococcus aureus*; ^b^ **MRSE:** Methicillin resistant *Staphylococcus epidermidis*.

**TABLE 1B:** Resistance profile of *E. coli* and *K. pneumoniae* ESBL producers.

Antimicrobial drug	***E. coli* ESBL** ^a^ **resistance (n = 9) (%)**	***K. pneumoniae* ESBL resistance (n=15) (%)**
Ceftriaxone	9 (100%)	15 (100%)
Cefepime	9 (100%)	12 (80%)
Ciprofloxacin	5 (55.5%)	12 (80%)
Gentamicin	4 (44.4%)	6 (40%)
Meropenem	0	0
Imipenem	0	0
Piperacillin-tazobactam	0	0

^a^ **ESBL:** Extended spectrum beta lactamase.

The association analysis showed that younger patients were more likely to harbor BSI-MRSA compared to older patients (≥45 years of age), and this was statistically significant (OR:0.28; CI95%:0.07-0.98; *p*=0.02). Our results also showed a trend among PLWHA who tended to develop BSI-MRSA infections. In addition, patients with BSI-MRSA had a lower chance of remaining in outpatient care compared to those with BSI-MSSA (OR:0.21, 95CI:0.06 to 0.66, *p*=0.003) (Table 2). 

**TABLE 2:** Overview of bloodstream culture samples from 53 patients in Manaus, detailing organisms with and without methicillin-resistant *Staphylococcus aureus*, from January 2017 to December 2019.

General Data	**Methicillin-resistant *Staphylococcus aureus***	**Methicillin-sensible *Staphylococcus aureus***	Odds Ratio	95% CI
	N=24 (%)	N=36 (%)		
**Male**	16 (66.7)	19 (52.7)	0.55	0.19-1.63
**Female**	8 (33.3)	17 (47.3)		
**Age (years)**				
0-18	5 (20.8)	5 (13.8)	1.63	0.41-6.38
19-44	15 (62.5)	16 (44.5)	2.12	1.00-13.39
≥45	4 (16.6)	15 (41.6)	0.28	0.07-0.98
**Associated comorbidities**				
HIV/AIDS	13 (54.1)	11 (30.5)	2.29	0.75-6.91
Pneumopathy	6 (25.0)	5 (13.8)	2.06	0.55-7.74
Nephropathy	3 (12.5)	5 (13.8)	0.88	0.19-4.11
Dermatopathy	11 (45.8)	14 (38.8)	1.32	0.46-3.78
**Length of hospital stay**				
0-7 days	2 (11.1)	4 (14.2)	0.75	0.12-4.58
>7 days	16 (88.8)	25 (85.7)	0.75	0.08-4.76
**Clinical outcome**				
Death	13 (54.1)	11 (30.5)	2.68	0.92-7.84
Hospital discharge	4 (16.6)	3 (8.3)	2.20	0.44-10.8
Outpatient care	6 (25.0)	22 (61.1)	0.21	0.06-0.66

Surveillance of BSI is essential for controlling the emergence of treatment-resistant microorganisms. Resistant pathogens have been widely reported in Brazil, and some cases have been found in Manaus city^7^. In this study, we evaluated common pathogen isolates and AMR patterns of blood cultures from a tertiary hospital in Brazil. 

The emergence of MRSA is problematic, underscoring the importance of monitoring and identifying circulating bacterial phenotypes^8^. Among all*S. aureus*BSI in our hospital, the MRSA frequency was 40%. Although our results show high rates of MRSA, a 20-year analysis of the SENTRY Antimicrobial Surveillance Program, of which Brazil is a participant, showed a decrease in BSI-MRSA occurrence, mainly between 2013-2016 (~30.0%), below the frequency found in our study^9^. 

Fortunately, the MRSA isolates in this study were highly susceptible to vancomycin and linezolid. Thus, while fluoroquinolones are agents that can be used to treat MRSA infections, resistance rates to this class of antimicrobial agents have increased over the years due to genetic mutations^10^. In our study, we found high rates of ciprofloxacin and fluoroquinolone resistance among the MRSA isolates (66.6%). The increase in this resistance may be related to the higher frequency of prescription of this class of antimicrobial agents.

MRSA is the most common cause of BSI in PLWHA and typically results in more severe outcomes for immunocompromised individuals, correlating with increased mortality rates^11^. Our results similarly suggest a propensity among PLWHA patients to develop BSI-MRSA. We speculate that the lack of statistical significance in this data (*p*=0.07) might be attributed to our small sample size. Research has indicated that HIV infection is a predictor of 30-day mortality in patients with BSI-MRSA^12^. Although death was not significant in people with BSI-MRSA (*p=0.11*), we observed that nine PLWHA with BSI-MRSA died. 

The emergence of MRSE is a concern because it is associated with BSI in patients who use medical devices such as central venous catheters. The frequency of MRSE in this study was 9.5%, which was far lower than that reported in a study that showed *S. epidermidis* in 95.5% of CoNS infections. Among them 97.5% of the isolates showed resistance to methicillin, 79.5% to ciprofloxacin and sulfamethoxazole-trimethoprim, and 71.8% to erythromycin and gentamicin, which is similar to the results obtained in our study^13^.

More than 5% of BSI cases are caused by *E. coli* and *K. pneumoniae,* often manifesting as secondary infections, or in association with intensive care unit admission^14^. Within the ESBL-producing isolates, we observed an increased resistance to cefepime (87.5%) and ciprofloxacin (70.8%) compared to non-ESBL producers. We did not observe any isolates resistant to carbapenems, nor did we identify MDR strains. Furthermore, a study on nosocomial infections in China showed a decrease in the isolation of resistant *K. pneumoniae* during 2015-2016 in BSIs, which was not observed for *E. coli*. In contrast, an increased incidence of third-generation cephalosporin-resistant *K. pneumoniae* was found, similar to that observed in our study^15^.

This study is limited by its observational and retrospective nature, as patient selection may have influenced data interpretation. Further, given that we analyzed the blood cultures of patients treated at a tertiary health unit, the results may not apply to other patient populations. The data described in this study were extracted from a database of electronic medical records; therefore, some data could not be included due to unavailability. In addition, this lack of information in medical records made it difficult to classify BSI as community- or healthcare-associated.

In conclusion, our study provides information on the frequency of bacterial pathogens in BSIs and their corresponding AMR patterns at a tertiary hospital in the Amazon region. We observed high rates of AMR among MRSA, MRSE, and ESBL-producing strains. In addition, MRSA isolates were susceptible to vancomycin and linezolid, and ESBLs to carbapenems. This research stands as the first study reporting on the local epidemiology of BSI at this center. Our findings underscore the importance of persistent vigilance and rational use of antimicrobial agents. They also enrich our understanding of the hospital’s microbiome and pave the way for more targeted therapeutic interventions for patients. Furthermore, this study provides preliminary information for future studies into the evolution and patterns of microorganisms that cause bloodstream infections.
